# Effects of continuous positive airway pressure on plasma fibrinogen levels in obstructive sleep apnea patients: a systemic review and meta-analysis

**DOI:** 10.1042/BSR20203856

**Published:** 2021-01-29

**Authors:** Juan Lin, Suxian Hu, Yonghong Shi, Fang Lu, Wen Luo, Yihua Lin

**Affiliations:** 1 Department of Respiratory and Critical Care Medicine, The First Affiliated Hospital of Xiamen University, Xiamen, Fujian, China; 2 Department of Clinical Medicine, Fujian Medical University, Fuzhou, Fujian, China

**Keywords:** continuous positive airway pressure, meta analysis, obstructive sleep apnea, plasma fibrinogen

## Abstract

***Objective:*** Fibrinogen has been implicated to play a role in the pathophysiology of obstructive sleep apnea (OSA). Many studies have evaluated the effect of continuous positive airway pressure (CPAP) on plasma fibrinogen levels in OSA patients. However, results from different reports were not consistent. To assess the effect of CPAP treatment on plasma fibrinogen levels of patients with OSA, a meta-analysis was performed.

***Methods:*** A systematic search of Pubmed, Embase, Cochrane, Wanfang Database and Chinese National Knowledge Infrastructure was performed. Data were extracted, and then weighted mean difference (WMD) and 95% confidence intervals (CIs) were calculated using a random-effects model.

***Results:*** Twenty-two studies involving 859 patients were included in this meta-analysis. Combined data showed that plasma fibrinogen concentrations decreased after CPAP therapy (WMD = −0.38 g/l, 95% CI [−0.54 to −0.22 g/l], *P*<0.001). In the subgroup analyses by therapy duration, plasma fibrinogen concentrations declined significantly in the long-term (≥1 month) CPAP therapy subgroup (WMD = −0.33 g/l, 95% CI [−0.49 to −0.16 g/l], *P*<0.001) but not in the short-term (<1 month) CPAP therapy subgroup (WMD = −0.84 g/l, 95% CI [−1.70 to 0.03 g/l], *P*=0.058). Moreover, in patients with long-term CPAP therapy duration, plasma fibrinogen levels decreased with good CPAP compliance (≥4 h/night) (WMD = −0.37 g/l, 95% CI [−0.55 to −0.19 g/l], *P*<0.001) but not with poor CPAP compliance (<4 h/night) (WMD = 0.12 g/l, 95% CI [−0.09 to 0.33 g/l], *P*=0.247).

***Conclusion*:** Long-term CPAP treatment with good compliance can reduce the plasma fibrinogen levels in patients with OSA.

## Introduction

Obstructive sleep apnea (OSA), currently characterized by the presence of recurrent episodes of partial or complete upper-airway collapse during sleeping, is a highly prevalent sleep disorder in developed countries [1]. Emerging data showed that patients with OSA have increased risks for cardiovascular diseases (CVDs) [2], which are associated with increased OSA mortalities [3].

The prothrombotic state is one of the important pathways connecting OSA with CVD [4]. The plasma fibrinogen plays a key role in thrombogenesis [5]. According to the Fibrinogen Studies Collaboration, a long-term increase of 1 g/l in fibrinogen level is associated with an approximate doubling risk of major CVDs in a wide range of circumstances in healthy middle-aged adults [6]. Moreover, the plasma fibrinogen concentrations are elevated in patients with OSA and associated with the severity of OSA [7–9]. So the elevated fibrinogen levels may be an important link between OSA and CVD.

Continuous positive airway pressure (CPAP), which eliminates arterial oxyhemoglobin desaturation and hypercapnia [10], is the most effective treatment of OSA [11]. Therapy with CPAP is associated with significant benefits to cardiovascular morbidity and mortality in OSA patients [12]. Moreover, CPAP has displayed remarkable values on improving hypercoagulability in OSA patients [13]. Several studies have assessed the effect of CPAP treatment on circulating fibrinogen concentrations in OSA patients. However, results from different reports were not consistent. Meanwhile, some studies were conducted on a small size, and therefore may not be able to provide sufficient evidence. In order to better evaluate the effects of CPAP on plasma fibrinogen levels in OSA patients, a systematic review and meta-analysis was performed.

## Methods

### Literature sources and search strategy

Two investigators (Juan Lin and Suxian Hu) searched PubMed, Embase, Cochrane, Wanfang Database and Chinese National Knowledge Infrastructure (CNKI) databases up to 11 October 2020 to identify potentially relevant articles independently. Disagreements were resolved via discussion or adjudicated by a third author (Yihua Lin). The following search terms were used: (‘sleep apnea, obstructive’ [Mesh] OR ‘sleep apnea syndromes’ [Mesh] OR ‘obstructive sleep apnoea’ [Title/Abstract] OR ‘obstructive sleep apnea’ [Title/Abstract]) and (‘fibrinogen’ [Title/Abstract] OR ‘fibrinogen’ [Mesh]) and (‘CPAP’ [Mesh] OR ‘CPAP’ [Title/Abstract] OR ‘continuous positive airway pressure’ [Title/Abstract] OR ‘positive end expiratory pressure’ [Title/Abstract]). The language of publication was English or Chinese. References of all selected articles were retrieved to identify other relevant studies.

### Inclusion and exclusion criteria

The studies were included if they met the following items:

1. All participants included in the study were adults.
2. The diagnosis of OSA was apnea hypopnea index (AHI) ≥ 5 events/h by full-night polysomnography.
3. The subjects did not receive any treatment before.
4. All subjects underwent CPAP treatment in the study.
5. Plasma concentrations of fibrinogen in the subjects were measured before and after CPAP treatment.

The studies were excluded if they met the following items:

1. Not enough original data.
2. Conferences or case reports.
3. The article was not written in English or Chinese.

### Quality assessment

In order to assess the methodological quality of included studies, articles were classified according to levels of evidence (LOE) by using criteria developed by DynaMed’s evidence-based methodology for interventional conclusion [14].

1. Level 1 (likely reliable) evidence represents research results that address clinical outcomes and meet an extensive set of quality criteria that minimizes bias.
2. Level 2 (mid-level) evidence represents research results that address clinical outcomes and demonstrate some methods of scientific investigation but do not meet the quality criteria to achieve Level 1.
3. Level 3 (lacking direct) evidence represents either of the following:
  - Reports that are not based on scientific analysis of clinical outcomes (e.g., case series, case reports, conclusions extrapolated indirectly from scientific studies).
  - Research results that do not address clinical outcomes, regardless of the scientific rigor.

### Data extraction

The data were extracted independently by two investigators (Juan Lin and Suxian Hu) and a consensus was reached on all items. Disagreements were resolved as described above. The following information was extracted: first author, year of publication, country, sample size, body mass index (BMI), gender, age, comorbidities, AHI, severity of OSA, therapy duration, CPAP time (h/night), plasma fibrinogen levels before and after CPAP treatment displayed by mean and standard deviation (SD). Standard error of mean (SEM) was transformed into SD using statistical formulas [15].

### Statistical analysis

The data were analyzed using STATA (Stata Corp. 2015. Stata Statistical Software: Release 14. College Station, TX: Stata Corp LP.). The effects of CPAP treatment on plasma fibrinogen concentrations in OSA patients were evaluated. *I*^2^ values were used to quantify heterogeneity [16]. The random-effects model was adopted if *P*≤0.10 or *I*^2^ ≥ 50%, which indicated that the included studies were moderately or highly heterogeneous; otherwise, the fixed-effects model was used. Sensitivity analysis and subgroup analysis was performed to identify the reason of heterogeneity [17]. Egger’s test, Begg’s test and funnel plot were used to assess the publication bias [18].

## Results

### Study inclusion and characteristics

**Figure 1:**
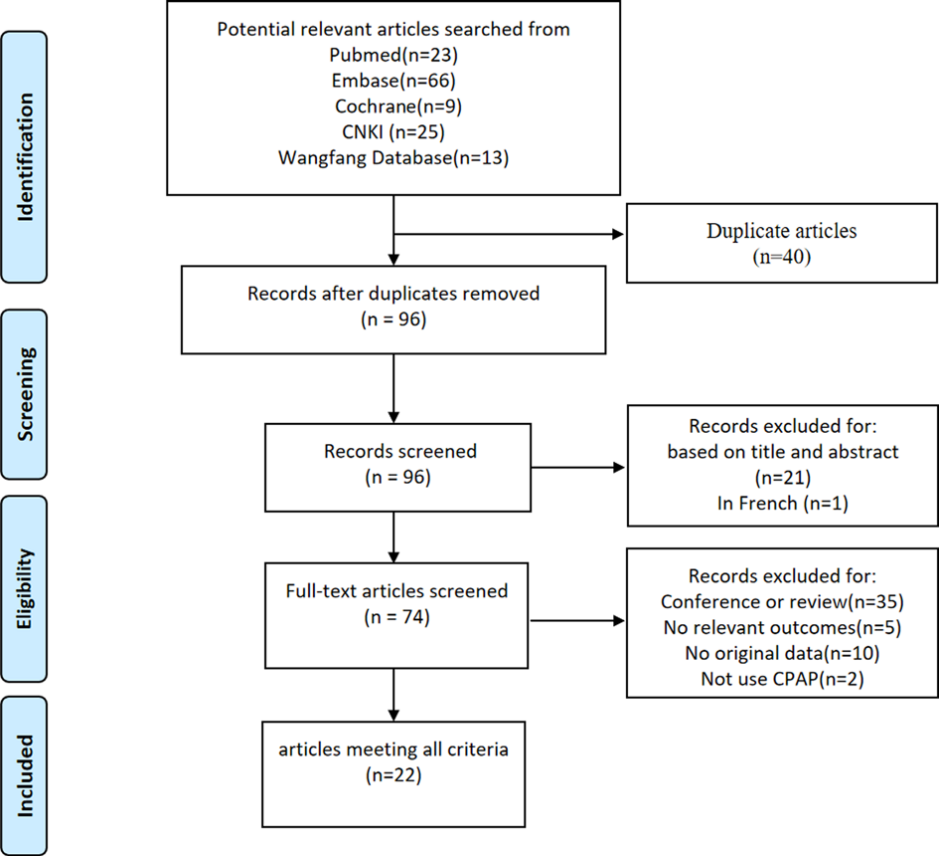
Flow of study identification, inclusion and exclusion

The procedure for identifying and selecting eligible studies is shown in Figure 1. A total of 136 articles were retrieved after initial search. Among them, 40 duplicate records were removed, leaving 96 papers for screening. Twenty-one records were excluded based on titles and abstracts and one paper was excluded for being in French. And then 52 records were excluded after full-text review for various reasons. Among them, 35 papers were excluded for conference or review, 5 papers had no relevant outcomes, 10 papers had not enough original data, and 2 papers did not use CPAP. Finally, 22 articles were included for this meta-analysis, containing 859 OSA patients in total [19–40]. Among them, 16 articles were from China [19,21,26–38,40]. In the included studies, the severity of OSA contained mild to moderate [32,38], moderate to severe [19,22,23,26,27,29–32,34,36,37,39], severe [20,21,24] and uncategorized [25,28,33,35,40]. The duration of CPAP therapy in eligible studies was categorized into short-term (<1 month) [19,23,33,40] and long-term (≥1 month) [19–22,24–32,34–39]. In the long-term CPAP treatment group, the patients received CPAP treatment for different hours every night, which were divided into good compliance (≥4 h/night) [19–22,24–31,34–39], poor compliance (<4 h/night) [20,21,39] and no mention of time [32]. In the short-term CPAP treatment group, the patients received CPAP treatment for different hours every night, which were also divided into good compliance (≥4 h/night) [19,23,40] and no mention of time [33]. In terms of comorbidities, 4 articles recruited OSA patients with comorbidities [21,23,29,32], 15 articles recruited OSA patients without comorbidities [19,20,24,27,28,30,31,33–40], 1 article recruited both patients with comorbidities and patients without comorbidities [25], and the remaining 2 articles did not mention patients’ comorbidities [22,26]. According to the severity of OSA, the term of CPAP treatment, the compliance of CPAP treatment and comorbidities, 22 articles were divided into 32 studies. All studies had an LOE score of 2. The main characters of all the included studies are presented in Tables 1 and 2.

**Table 1:** Characteristics of studies included in the meta-analysis

Study (first author, year)	Country	Sample size	BMI (kg/m^2^)	Gender	Age	Comorbidities	LOE
Chin K., 1996	Japan	11	31.10 ± 5.30	ND	46.20 ± 12.60	Two hypertension patients	2
Chin K., 1998	Japan	15	31.10 ± 5.03	15 males	45.30 ± 12.00	ND	2
Du X., 2003	China	38	28.80 ± 2.10	34 males, 4 females	50.40 ± 10.80	Exclude chronic disease	2
Su M., 2003	China	13	26.40 ± 3.20	10 males, 3 females	51.00 ± 6.30	Exclude chronic disease	2
Zhang X., 2003	China	41	26.90 ± 3.00	29 males, 12 females	63.40 ± 4.00	ND	2
Zhang X., 2003-1a	China	16	27.00 ± 3.00	12 males, 4 females	41.00–65.00	Exclude chronic disease	2
Zhang X., 2003-1b	China	16	27.00 ± 3.00	12 males, 4 females	41.00–65.00	Exclude chronic disease	2
Zhang X., 2003-2a	China	16	26.70 ± 3.10	12 males, 4 females	64.00 ± 4.10	Exclude chronic disease	2
Zhang X., 2003-2b	China	16	26.70 ± 3.10	12 males, 4 females	64.00 ± 4.10	Exclude chronic disease	2
Zhang X., 2004	China	16	26.7 ± 3.100	12 males, 4 females	50.00 ± 6.00	Exclude chronic disease	2
Dorkova Z., 2008a	Slovak Republic	16	32.80 ± 4.40	15 males, 1 female	51.30 ± 9.50	Exclude chronic disease	2
Dorkova Z., 2008b	Slovak Republic	15	37.30 ± 6.90	10 males, 5 females	56.10 ± 9.50	Exclude chronic disease	2
Chen X., 2010	China	40	26.80 ± 4.30	27 males, 13 females	47.40 ± 5.90	Exclude chronic disease	2
Feng J., 2010	China	30	ND	23 males, 7 females	54.30 ± 11.60	Exclude chronic disease	2
Kumor M., 2011a	Poland	16	30.40 ± 3.80	ND	54.20 ± 6.90	With IHD	2
Kumor M., 2011b	Poland	24	30.30 ± 2.80	ND	50.00 ± 9.80	Exclude chronic disease	2
Zhang X., 2011a	China	15	ND	13 males, 2 females	55.00–75.00	Exclude chronic disease	2
Zhang X., 2011b	China	15	ND	13 males, 2 females	55.00–75.00	Exclude chronic disease	2
Zhang X., 2011c	China	15	ND	13 males, 2 females	55.00–75.00	Exclude chronic disease	2
Xu D., 2012	China	85	ND	ND	31.00–55.00	Exclude chronic disease	2
Ni L., 2014a	China	24	30.40 ± 3.80	ND	ND	Exclude chronic disease	2
Ni L., 2014b	China	16	30.30 ± 2.80	ND	ND	Exclude chronic disease	2
Xu J., 2014a	China	33	31.82 ± 5.60	ND	ND	13 hypertension, 6 diabetes	2
Xu J., 2014b	China	17	36.50±4.36	ND	ND	9 hypertension, 5 diabetes	2
Zhang X., 2014a	China	34	ND	ND	ND	With IHD	2
Zhang X., 2014b	China	11	ND	ND	ND	With IHD	2
Wang H., 2015	China	48	ND	ND	ND	With IHD	2
Kisabay A., 2016	Turkey	65	29.43 ± 2.33	53 males,12 females	48.23 ± 11.17	Exclude chronic disease	2
Kimihiko M., 2020a	Japan	27	27.50 ± 4.10	23 males,4 females	60.00 ± 9.00	Exclude chronic disease	2
Kimihiko M., 2020b	Japan	33	28.50 ± 4.30	28 males, 5 females	56.00 ± 11.00	Exclude chronic disease	2
Wang Z., 2020	China	40	25.81 ± 1.16	24 males, 16 females	52.37 ± 6.14	Exclude chronic disease	2
Xu Y., 2020	China	42	ND	26 males, 16 females	58.71 ± 8.34	Exclude chronic disease	2

Abbreviations: IHD, ischemic heart disease; ND, no data.

**Table 2:** The characteristics of studies included in the meta-analysis

Study (first author, year)	AHI	Severity	CPAP duration	Daily duration (h/night)	Fibrinogen levels (mean ± SD)	
					Pre-CPAP (g/l)	Post-CPAP (g/l)
Chin K., 1996	62.90 ± 20.90	Moderate to severe	1 day	>6	2.98 ± 0.53	2.76 ± 0.37
Chin K., 1998	61.50 ± 16.30	Moderate to severe	6 months	≥5	2.99 ± 0.62	2.92 ± 0.66
Du X., 2003	56.00 ± 22.00	Mild to severe	1 day	ND	5.43 ± 1.59	3.98 ± 1.37
Su M., 2003	≥15	Moderate to severe	1 month	6–8	2.75 ± 0.13	2.74 ± 0.14
Zhang X., 2003	37.40 ± 9.60	Moderate to severe	1 month	6–8	2.96 ± 0.14	2.77 ± 0.15
Zhang X., 2003-1a	≥15	Moderate to severe	1 month	6–8	2.77 ± 0.12	2.75 ± 0.13
Zhang X, 2003-1b	≥15	Moderate to severe	1 month	6–8	2.97 ± 0.12	2.76 ± 0.12
Zhang X., 2003-2a	39.90 ± 11.50	Moderate to severe	1 month	6–8	2.80 ± 12.00	2.80 ± 13.00
Zhang X., 2003-2b	39.90 ± 11.50	Moderate to severe	1 month	6–8	3.00 ± 12.00	2.70 ± 12.00
Zhang X., 2004	≥15	Moderate to severe	1 month	>6	2.97 ± 0.12	2.76 ± 0.12
Dorkova Z., 2008a	64.70 ± 23.30	Severe	2 months	≥4	3.16 ± 0.99	3.45 ± 0.89
Dorkova Z., 2008b	63.20 ± 18.80	Severe	2 months	<4	3.43 ± 0.68	3.46 ± 0.93
Chen X., 2010	≥5	Mild to severe	1 month	6–8	3.87 ± 0.51	2.43 ± 0.26
Feng J., 2010	55.10 ± 10.20	Moderate to severe	6 months	6–8	3.95 ± 0.75	3.21 ± 0.45
Kumor M., 2011a	48.30 ± 20.30	Mild to severe	3 months	4.40 ± 1.80	3.50 ± 0.88	3.68 ± 1.05
Kumor M., 2011b	45.40 ± 21.50	Mild to severe	3 months	4.40 ± 1.80	3.11 ± 0.53	3.28 ± 0.71
Zhang X., 2011a	≥15	Moderate to severe	15 days	6–8	3.00 ± 0.58	2.89 ± 0.45
Zhang X., 2011b	≥15	Moderate to severe	1 month	6–8	3.00 ± 0.58	2.68 ± 0.54
Zhang X., 2011c	≥15	Moderate to severe	3 months	6–8	3.00 ± 0.58	2.22 ± 0.33
Xu D., 2012	≥15	Moderate to severe	1 month	6–8	6.20 ± 1.10	3.40 ± 0.70
Ni L., 2014a	48.30 ± 20.30	Mild to severe	3 months	5.20 ± 1.50	3.50 ± 0.88	3.68 ± 1.05
Ni L., 2014b	45.40 ± 21.50	Mild to severe	3 months	4.40 ± 1.80	3.11 ± 0.54	3.28 ± 0.71
Xu J., 2014a	64.40 ± 16.00	Severe	10 weeks	≥4	3.12 ± 0.68	3.29 ± 0.61
Xu J., 2014b	63.40 ± 15.40	Severe	10 weeks	<4	3.23 ± 0.58	3.45 ± 0.40
Zhang X., 2014a	≥15	Moderate to severe	2 months	ND	3.53 ± 0.97	2.99 ± 0.82
Zhang X., 2014b	<15	Mild	2 months	ND	2.40 ± 0.56	2.10 ± 0.46
Wang H., 2015	≥15	Moderate to severe	1 month	8	2.94 ± 0.28	1.87 ± 0.36
Kisabay A., 2016	>30	Severe	3 months	5.60	3.45 ± 0.76	3.15 ± 0.49
Kimihiko M., 2020a	39.20 ± 25.70	Moderate to severe	3 months	5.70 ± 0.80	2.75 ± 0.46	2.55 ± 0.47
Kimihiko M., 2020b	43.30 ± 28.89	Moderate to severe	3 months	3.10 ± 1.00	2.77 ± 0.52	2.84 ± 0.70
Wang Z., 2020	35.26 ± 4.36	Mild to moderate	1 month	≥5	3.03 ± 0.33	2.67 ± 0.29
Xu Y., 2020	26.23 ± 7.25	Mild to severe	2 weeks	≥4	4.53 ± 0.66	2.93 ± 0.47

Abbreviation: ND, no data.

### Quantitative analysis

**Figure 2:**
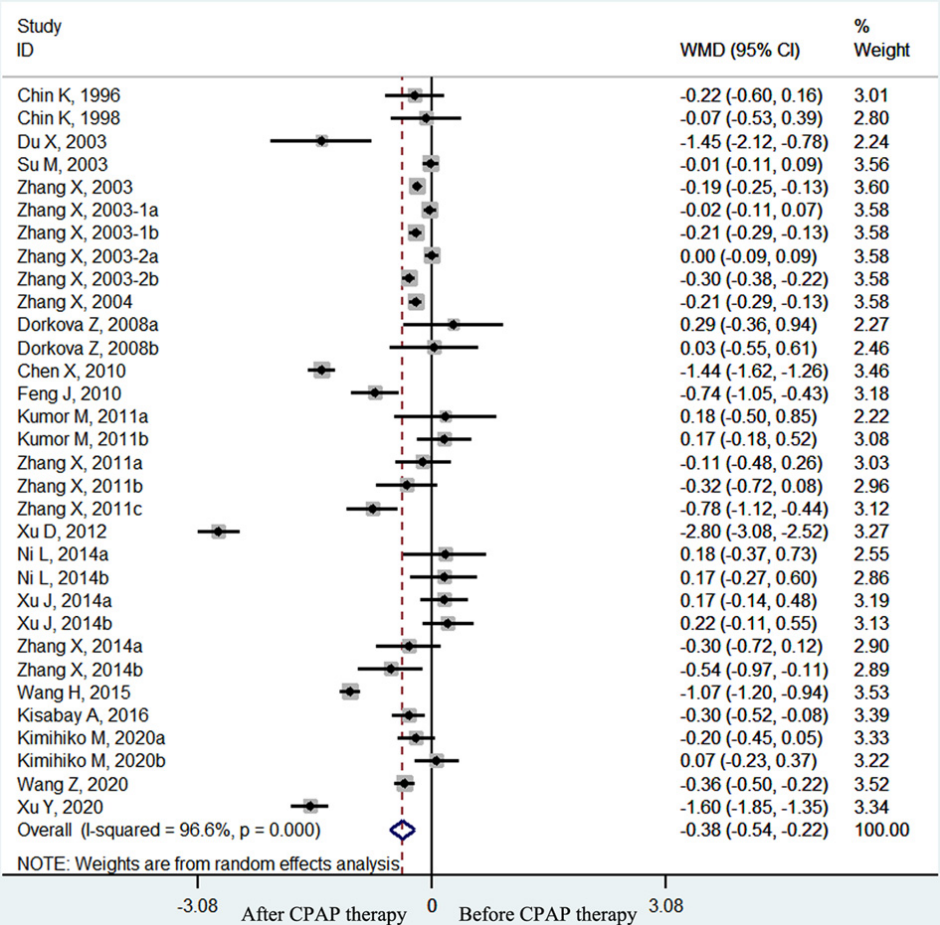
The forest plot of plasma fibrinogen concentrations before and after CPAP treatment in OSA patients

**Figure 3:**
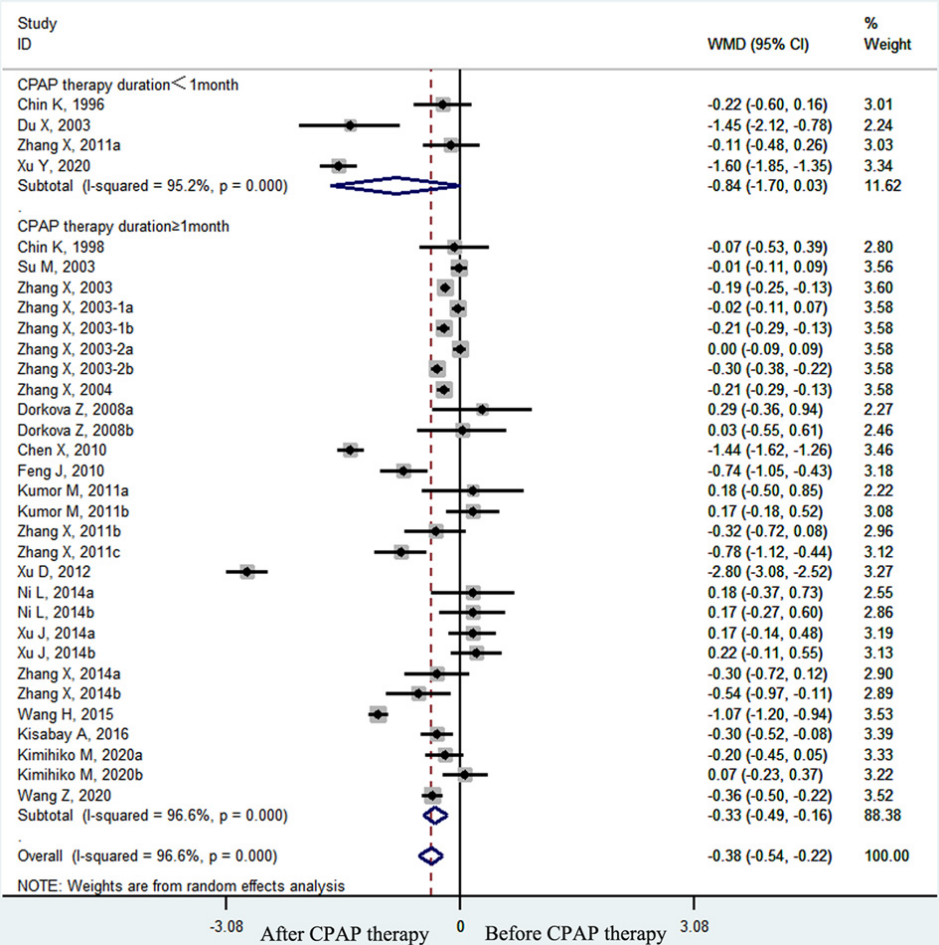
The forest plot of plasma fibrinogen concentrations in OSA patients with long-term CPAP therapy duration (≥1 month) or short-term CPAP therapy duration (<1 month)

**Figure 4:**
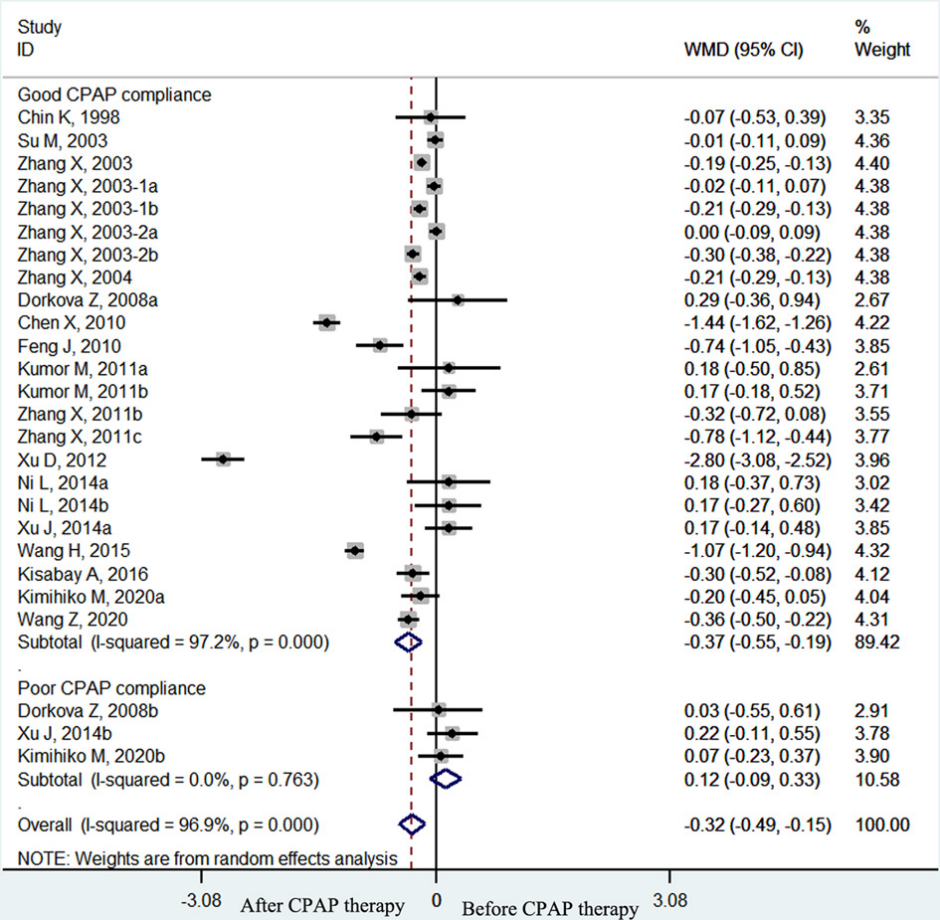
The forest plot of plasma fibrinogen concentrations in OSA patients with good CPAP compliance compared with those with poor CPAP compliance in long-term CPAP therapy

**Figure 5:**
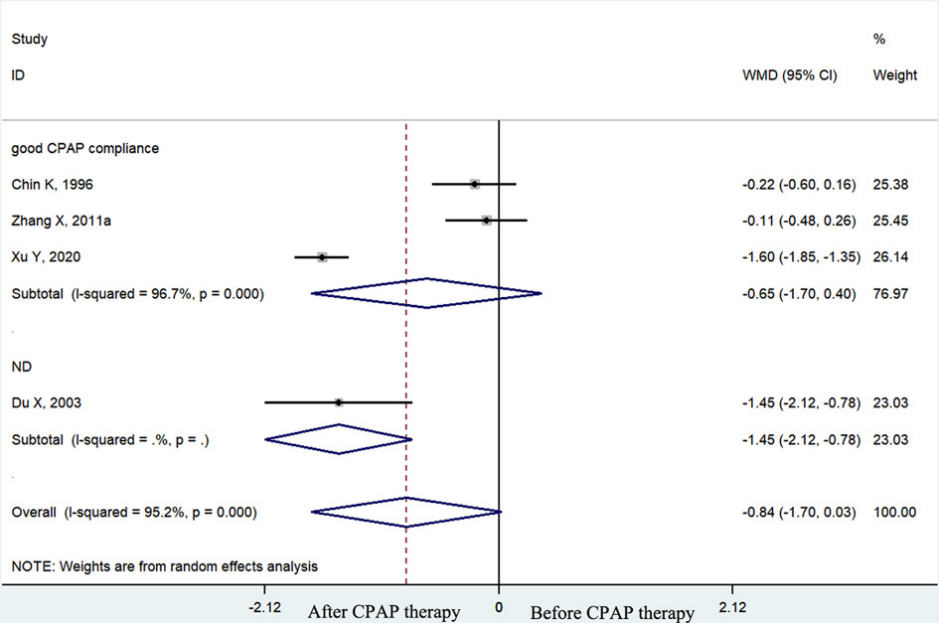
The forest plot of plasma fibrinogen concentrations in OSA patients with good CPAP compliance compared with those who did not mention the time in short-term CPAP therapy

**Figure 6:**
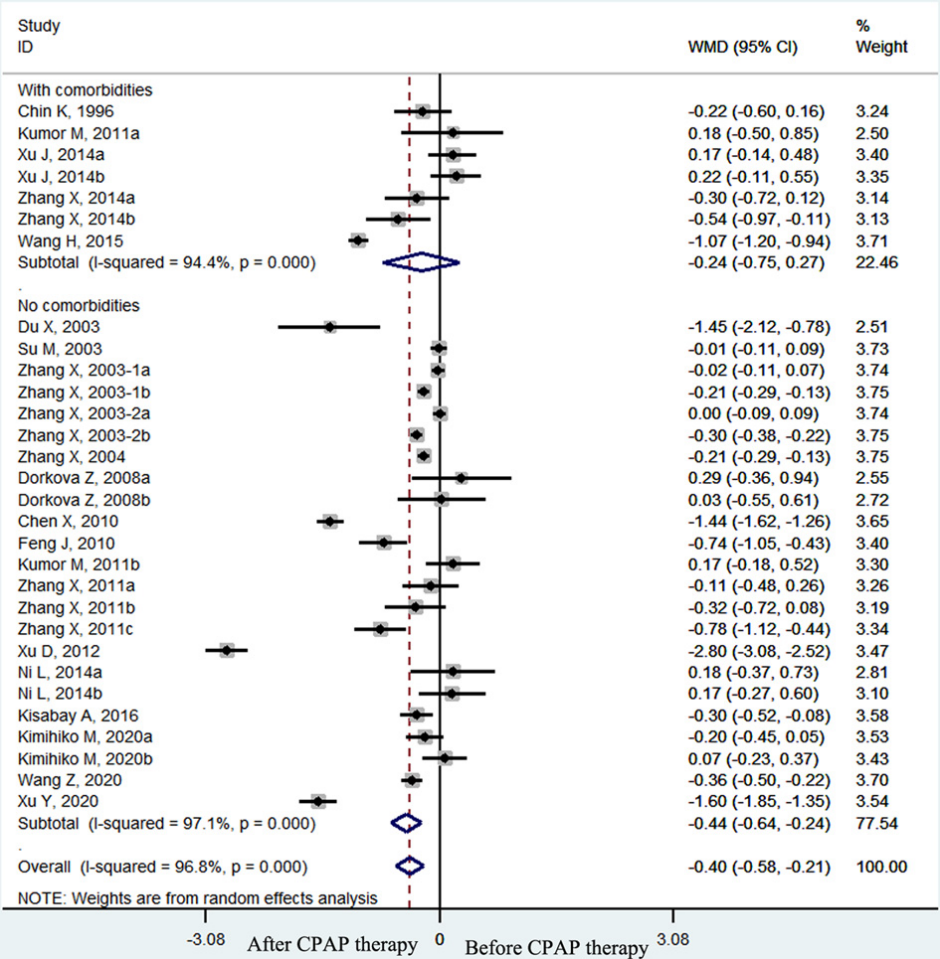
The forest plot of plasma fibrinogen concentrations before and after CPAP treatment in OSA patients with or without comorbidities

The pooled effect size showed that the plasma fibrinogen concentrations of OSA patients decreased significantly after CPAP treatment (weighted mean difference (WMD) = −0.38 g/l, 95% confidence interval (CI) [−0.54 to −0.22 g/l], *P*<0.001; *I*^2^ = 96.6%, *P*<0.001; Figure 2). In the subgroup analyses by therapy duration, plasma fibrinogen concentrations declined significantly in the long–term (≥1 month) CPAP therapy subgroup (WMD = −0.33 g/l, 95% CI [−0.49 to −0.16 g/l], *P*<0.001; *I*^2^ = 96.6%, *p* < 0.001; Figure 3) but not in the short-term (< 1 month) CPAP therapy subgroup (WMD = −0.84 g/l, 95% CI [−1.70 to 0.03 g/l], *P*=0.058; *I*^2^ = 95.2%, *P*<0.001; Figure 3). Moreover, in OSA patients treated with long-term CPAP, fibrinogen level decreased only in patients with good CPAP compliance (≥4 h/night) (WMD = −0.37 g/l, 95% CI [−0.55 to −0.19 g/l], *P*<0.001; *I*^2^ = 97.2%, *P*<0.001; Figure 4), but not in those with poor CPAP compliance (<4 h/night) (WMD = 0.12 g/l, 95% CI [−0.09 to 0.33 g/l], *P*=0.247; *I*^2^ = 0.0%, *P*=0.763; Figure 4). In the short-term CPAP treatment group, fibrinogen level did not decrease significantly, even in those with good CPAP compliance (WMD = −0.65 g/l, 95% CI [−1.70 to 0.40 g/l], *P*=0.223; *I*^2^ = 96.7%, *P*<0.001; Figure 5). In another subgroup analyses by comorbidities, plasma fibrinogen concentrations declined significantly in patients without comorbidities (WMD = −0.44 g/l, 95% CI [−0.58 to −0.21 g/l], *P*<0.001; *I*^2^ = 96.8%, *P*<0.001; Figure 6) but not in patients with comorbidities (WMD = −0.24 g/l, 95% CI [−0.75 to 0.27 g/l], *P*=0.364; *I*^2^ = 94.4%, *P*<0.001; Figure 6).

### Sensitivity analysis

We performed sensitivity analyses for all the results. The observed significant results were not materially altered after we sequentially excluded each study.

### Publication bias

**Figure 7:**
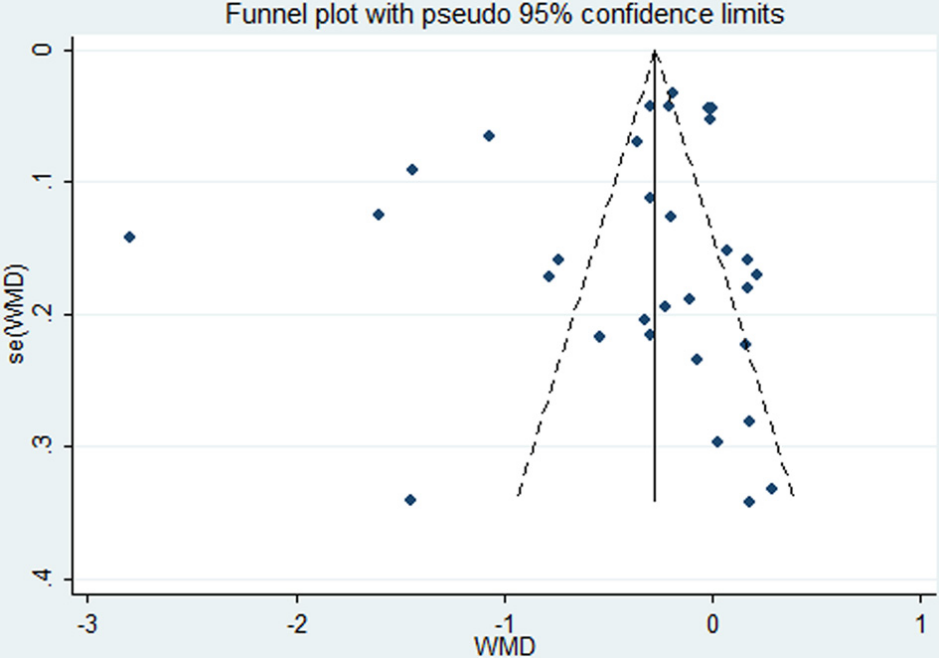
Funnel plots for evaluation of publication bias in the included studies

The funnel plot seemed unsymmetrical (Figure 7). However, Begg’s test (*P*=0.638) and Egger’s test (*P*=0.231) demonstrated that there was no evidence to confirm publication bias in the study. Moreover, after excluding the five studies [29,30,33,35,40] which deviated the furthest, the conclusions remained the same.

## Discussion

The present study conducted a systemic review and meta-analysis to investigate the efficacy of CPAP on fibrinogen levels in patients with OSA. To the best of our knowledge, this is the first systematic review and meta-analysis to assess the effect of CPAP treatment on plasma fibrinogen in OSA patients. The important finding of the present study is that long-term CPAP treatment with good compliance can reduce the plasma fibrinogen levels in patients with OSA.

In OSA patients, fibrinogen levels are elevated [41,42], even after adjusting for comorbidities such as arterial hypertension or coronary artery disease [43]. Moreover, the concentration of fibrinogen has been found to be directly related to severity of OSA patients [8]. However, the exact mechanism related to OSA and fibrinogen was not fully clear. It is well known that OSA is characterized by chronic persistent hypoxia, chronic intermittent hypoxia and sleep disturbance, which is related to increased reactive oxygen species (ROS) and oxidative stress response. Fibrinogen, which is in response to inflammation and ROS, affects blood coagulation, hemorheology and platelet aggregation, and promotes the progress of atherosclerosis [44]. Plasma fibrinogen elevation in OSA patients may be due to the combined effects of OSA-induced disturbances in biochemical neurohumoral regulation, inflammation and metabolism [45].

CPAP treatment can improve OSA associated hypoxia condition and oxidative stress. It is now accepted as the standard and ‘first-line’ treatment in current management of OSA [10]. The results of our study showed that CPAP treatment can reduce the plasma fibrinogen levels in OSA patients, which is in accordance with the previous studies of Kisabay et al. [24] and Feng et al. [27]. In the subgroup analyses, long-term CPAP therapy with good compliance can reduce the plasma fibrinogen levels, while long-term CPAP therapy with poor compliance or short-term CPAP therapy did not. This result should be attributed to the fact that long-term CPAP therapy with good compliance can improve hypoxia condition and oxidative stress in OSA patients better than short-term CPAP therapy or poor compliance.

Reducing fibrinogen levels in OSA patients might have potential important clinical implications. Fibrinogen enhances thrombosis and atherosclerosis via its effects on platelet aggregation, blood rheology and endothelial-cell injury [5]. Meanwhile, fibrinogen and its degradation products may further damage blood vessels by stimulating smooth muscle proliferation and migration [46,47]. A systemic review has confirmed the significance of elevated fibrinogen for prediction of future cardiovascular risk even in the healthy, middle-aged population [48]. Hence fibrinogen has been acknowledged as an important biomarker for cardiovascular risk [43,49], and may be responsible for the high incidence of CVD in OSA patients [50]. Therefore, CPAP may reduce cardiovascular mortality in OSA patients by reducing fibrinogen levels, which is in accordance with the study of Aslan et al. [51]. So, our study provided a robust evidence that once OSA is diagnosed, a long-term CPAP therapy with good compliance should be applied as soon as possible.

However, in OSA patients with comorbidities, the levels of fibrinogen did not decrease significantly after CPAP treatment. This may be due to both comorbidities [6] and OSA [7] can lead to elevated plasma fibrinogen levels, while CPAP can only improve the pathological condition of OSA. Thus, in addition to CPAP therapy, extra pharmacological treatment may be needed to treat comorbidities in OSA patients.

Several limitations still existed in our research. The heterogeneity test showed that there was heterogeneity among the studies, because many confounding factors may coexist in OSA patients. We failed to explore the reason of heterogeneity by subgroup analysis based on CPAP therapy duration(<1 and ≥1 month), CPAP daily duration (<4 and ≥4 h/night) and comorbidities. Moreover, in most of the included studies, the severity of OSA were moderate to severe, we did not perform subgroup analysis based on OSA severity.

## Conclusion

This meta-analysis indicates that CPAP treatment, especially with long-term and good compliance, can reduce plasma fibrinogen concentrations in OSA patients.

## Data Availability

All the data used and/or analyzed during the current study can be obtained by contacting the corresponding author on reasonable request.

## Abbreviations

AHI: apnea hypopnea index
CI: confidence interval
CPAP: continuous positive airway pressure
CVD: cardiovascular disease
LOE: level of evidence
OSA: obstructive sleep apnea
ROS: reactive oxygen species
SD: standard deviation
WMD: weighted mean difference
